# A High-Precision Short-Term Photovoltaic Power Forecasting Model Based on Multivariate Variational Mode Decomposition and Gated Recurrent Unit-Attention with Crested Porcupine Optimizer-Enhanced Vector Weighted Average Algorithm

**DOI:** 10.3390/s25195977

**Published:** 2025-09-26

**Authors:** Jinxiang Pian, Xianliang Chen

**Affiliations:** School of Electrical and Control Engineering, Shenyang Jianzhu University, Shenyang 110168, China; chenxl@stu.sjzu.edu.cn

**Keywords:** PV power prediction, multivariate variational modal decomposition, vector weighted average algorithm, gated recurrent unit, attention mechanism

## Abstract

The increasing reliance on renewable energy sources, such as photovoltaic (PV) systems, is pivotal for achieving sustainable development and addressing global energy challenges. However, short-term power forecasting for distributed PV systems often faces accuracy limitations, hindering their efficient grid integration. To address this, a novel hybrid prediction model is proposed, combining multivariate variational mode decomposition (MVMD) with a gated recurrent unit (GRU) network, an attention mechanism (ATT), and an enhanced vector weighted average algorithm (cINFO). The MVMD first decomposes historical data to reduce volatility. The INFO algorithm is then improved by integrating the crested porcupine optimizer (CPO), forming the cINFO algorithm to optimize GRU-ATT hyperparameters. An attention mechanism is incorporated to accentuate key influencing factors. The model was evaluated using the DKASC Alice Springs dataset. Results demonstrate high predictive accuracy, with mean absolute error (MAE), root mean square error (RMSE), and coefficient of determination (R^2^) values of 0.0249, 0.0693, and 99.79%, respectively, under sunny conditions, significantly outperforming benchmark models. This confirms the model’s feasibility and superiority for short-term PV power forecasting.

## 1. Introduction

The growing conflict among the energy crisis, environmental pollution, and rising electricity demand has attracted global attention to the development and utilization of clean energy [1]. Among various alternatives, photovoltaic (PV) power generation has gained widespread adoption due to its advantages of safety, abundance, wide availability, and potential cost-effectiveness [2]. As reliance on PV systems continues to grow, they play a pivotal role in achieving sustainable development and addressing global energy challenges.

The term ‘distributed photovoltaic (DPV) system’ in this work refers to a small-to medium-scale, grid-connected solar power generation installation (typically ranging from several kilowatts to a few megawatts) that is deployed at or near the point of consumption, such as on residential, commercial, or industrial rooftops or facilities [3,4]. Unlike utility-scale PV plants, the power output of distributed PV systems is highly dependent on hyper-local weather conditions and is characterized by higher volatility and uncertainty, posing greater challenges for grid integration and management. This inherent intermittency underscores the critical need for accurate and reliable forecasting models specifically tailored for distributed PV generation, which is the primary focus of the current study.

With PV power generation capacity increasing, its effective power output has become highly random, fluctuating, and intermittent [5]. This increases the complexity of energy storage demand and grid management, which can affect the economic efficiency and reliability of power supply for users. In this context, forecasting PV power generation has increasingly become a key focus in the research and development of future power systems [6,7]. PV power forecasting for distributed PV systems can improve power management, improve system reliability, support grid balance, enhance investment decisions, and promote renewable energy integration, thereby improving economics and stability.

PV power forecasting is generally classified by temporal resolution into long-term, medium-term (ranging from one month to one year), short-term (0–72 h), and ultra-short-term (within 4 h) [8]. Among these, short-term forecasting is especially critical, as it supplies timely and precise information on output fluctuations that supports the control, dispatch, and operation of PV plants and the broader grid [9]. Consequently, accurate short-term forecasting has become a fundamental technology for ensuring secure grid integration and reliable system performance [10].

Physical, statistical, artificial intelligence, and hybrid approaches are the most commonly used forecasting methods [11]. Physical methods rely heavily on precise geographic information and accurate meteorological data, making them sensitive to disturbances [12]. In contrast, statistical methods require large amounts of historical PV output data—such as autoregressive moving average (ARMA), autoregressive integrated moving average (ARIMA) [13], Markov chains [14], and Kalman filters [15]—and generate forecasts by analyzing these historical records.

With big data and computer technology development, artificial intelligence methods based on machine learning models have been widely studied and successfully applied to PV power prediction [16]. Iheanetu, K. et al. [17] proposed a deep learning method that integrates weather forecast data with a Multilayer Perceptron (MLP) for short-term PV power prediction. Sheng, W. et al. [18] proposed a prediction model based on a Support Vector Machine (SVM) for estimating hourly PV power generation. Al-Dahidi, S. et al. [19] employed Extreme Learning Machines (ELM) for PV power prediction, demonstrating that this approach can moderately enhance forecasting accuracy with minimal computational overhead.

However, shallow machine learning methods like SVM and ELM are limited in capturing the deep nonlinear and dynamic characteristics of PV power data. In contrast, deep learning models excel at nonlinear mapping and feature representation, effectively addressing the shortcomings of shallow approaches [20]. The most crucial aspect of deep learning is the utilization of neural networks, including Convolutional Neural Networks (CNN) [21], Long Short-Term Memory Neural Networks (LSTM) [22], and Gated Recurrent Units (GRU) [23]. In a related study, M. S. Hossain et al. [22] utilized Long Short-Term Memory (LSTM) networks combined with synthetic weather forecasting to predict PV power generation, which significantly enhanced prediction accuracy. Their findings demonstrated that the LSTM model achieved a high level of accuracy in this task. In another study, Sodsong et al. [24] successfully developed a GRU model incorporating cascade structure for predicting PV power generation. The results showed remarkable efficacy, underscoring the potential of GRU in this field. Although the efficacy of deep learning in prediction is widely acknowledged, issues such as overfitting emerge during training. Additionally, the erratic nature of PV power generation can further complicate prediction. Consequently, an increasing number of scholars have turned to combined prediction models, integrating diverse models and optimization algorithms within a unified framework to leverage their respective strengths and enhance the model’s predictive precision.

To improve sample data quality and reduce the volatility of historical PV power, many researchers have incorporated signal decomposition algorithms into hybrid forecasting models. Commonly used methods include empirical mode decomposition (EMD) [25], ensemble EMD (EEMD) [26], complete ensemble EMD with adaptive noise (CEEMDAN) [27], and variational mode decomposition (VMD) [28]. To refine the dataset while preventing the disclosure of confidential information, the method employed by Li et al. [29] involved decomposing the power data into distinct components using Empirical Mode Decomposition (EMD) and subsequently applying neural networks for predictive modeling. However, in certain instances, EMD is susceptible to modal aliasing, which impairs the clarity of the decomposition results. Additionally, EMD may be susceptible to instability in the edge portion of the signal, which can also impact the quality of the decomposition. Wang et al. [30] employed the Ensemble Empirical Mode Decomposition (EEMD) method to decompose and reconstruct raw PV power data into high- and low-frequency sub-sequences for feature extraction. These were then fed into a Long Short-Term Memory (LSTM) model, whose hyperparameters were optimized via Bayesian Optimization (BO), significantly improving both prediction accuracy and stability. Although EEMD alleviates some limitations of EMD, it still has certain constraints. In the literature [31], a hybrid forecasting model combining VMD, Deep Belief Network (DBN), and Autoregressive Moving Average (ARMA) was proposed. VMD overcomes the limitations of EMD and EEMD by decomposing the time series into components of different frequencies, while DBN and ARMA predict the high- and low-frequency components, respectively, which are then reconstructed to generate forecasts. The results show that this approach achieves both high accuracy and reliability. To handle highly correlated features, Qian liu et al. [32] introduced a multimodal decomposition method, VMD-CEEMD-SSA. Subsequently, the decomposed results and meteorological factors were incorporated into a BiLSTM-CNN hybrid model for prediction, which yielded highly accurate results. This study demonstrated that employing multiple decomposition can effectively suppress inaccuracies in prediction outcomes. However, the generation of a greater number of subsequences as a consequence of this approach may potentially impact the overall efficiency of the model. Additionally, VMD is highly sensitive to parameter settings, necessitating a multitude of experiments to achieve optimal parameters, which may increase the complexity of its implementation.

Recent advances have further addressed cross-site robustness and data augmentation challenges. Na et al. (2025) [33] proposeda privacy-preserving deep federated learning method for ultra-short-term photovoltaic power forecasting, combining federated learning, Transformer autoencoders, and joint probability models to improve accuracy while protecting data privacy. Similarly, Ji et al. (2024) [34] integrated generative adversarial networks (GANs) to augment incomplete meteorological records, while ElRobrini et al. (2024) [35] reduced retraining costs across heterogeneous plants in diverse climates via transfer learning. These studies highlight the growing focus on scalable and data-efficient. As shown in Table 1, a comparative analysis of representative limitations in PV forecasting models is presented.

Based on the insights from the aforementioned studies, this paper proposes a short-term PV power hybrid prediction model combining multivariate variational mode decomposition (MVMD) with the enhanced INFO-GRU-ATT. Initially, the original data are decomposed by the MVMD methodology in order to facilitate the further exploration of the hidden features and structures within the series. Subsequently, the decomposed components are fed into the improved INFO hyper-parameterized configuration of GRU-ATT for prediction purposes. Ultimately, the predicted components are integrated to yield the final prediction results.

The primary concepts and contributions of this paper are as follows:

(1) The selection of MVMD, which is capable of processing multi-channel signals simultaneously, offers a more effective means of capturing the inherent characteristics of the signals in question. This approach enhances the decomposition ability, stability, and robustness of the system.
(2) Improvement of INFO Optimization Algorithm: In this paper, a new optimization algorithm is proposed by combining the INFO optimization algorithm with the crested porcupine optimizer optimization algorithm (CPO). In the update process of INFO algorithm, the defense mechanism of CPO is added in order to increase the population diversity and avoid falling into a local optimum. Combined with the dynamic population adjustment strategy of CPO, the algorithm can keep more solutions for global exploration in the early stage of optimization and gradually reduce the population size in the later stage to accelerate the convergence. In the local search stage, the adaptive weight updating mechanism of CPO is introduced to adaptively adjust the position of the solution according to the value of the objective function to improve the search efficiency of the algorithm.
(3) The introduction of an attention mechanism enables the model to focus on processing the most pertinent aspects of the input data, thereby enhancing the accuracy and generalizability of the model’s predictive capabilities.

The enhanced MVMD, INFO, and GTU-ATT are integrated to form a unified prediction model, which is then evaluated against alternative models to ascertain the efficacy of the proposed combined prediction approach.

The rest of this paper is organized as follows. Section 2 details MVMD, GRU, Attention and INFO and explains the implementation procedures of the MVMD-cINFO-GRU-ATT model. Section 3 presents specific modeling details. Section 4 presents two different experiments to compare the proposed model with other models and conducts extensive discussions.

## 2. Methodology

The selection of MVMD over conventional decomposition techniques (e.g., VMD, EEMD) is motivated by its inherent capacity for synchronized multichannel processing, which preserves cross-variable frequency alignment critical for capturing the spatiotemporal correlations in multi-source PV data—significantly enhancing decomposition stability under volatile conditions. GRU-ATT is adopted as the core predictor due to its gating mechanism that dynamically regulates information flow, resolving gradient issues inherent in RNNs while maintaining LSTM-equivalent modeling capabilities with faster training convergence; the integrated attention mechanism further prioritizes volatility-driven features during high-irradiance fluctuations. For hyperparameter optimization, CPO-enhanced INFO combines the vector-weighted averaging of INFO with CPO’s defense mechanism and adaptive population control, effectively escaping local optima through quills-based diversity preservation while accelerating convergence via dynamic solution-space pruning.

### 2.1. MVMD

Multivariate variational mode decomposition (MVMD) extends the variational mode decomposition (VMD) framework to handle multi-channel datasets. It first establishes a multivariate oscillation model, which is constructed on the basis of the shared or common frequency components across all input channels. By applying this model, a variational optimization problem is formulated with the objective of extracting a set of band-limited intrinsic modes that capture the underlying multivariate oscillations within the input signals.

Unlike applying VMD independently to each channel, MVMD directly identifies multivariate modulation oscillations in the multidimensional space where the signals coexist. In contrast, the channel-wise VMD approach simply decomposes each signal separately, yielding only univariate oscillations confined to individual channels in a one-dimensional space. Consequently, such an approach fails to capture cross-channel oscillations or any meaningful joint information among signals. In comparison, MVMD is able to recover multivariate oscillations from the data, which naturally leads to the property of pattern alignment—that is, aligning or matching frequency components shared by different channels [36]. To guarantee that the decomposed sequences remain consistent in both temporal and frequency domains, and to preserve the synchronization, correlation, and interdependence among multivariate components, MVMD introduces a joint frequency alignment mechanism across channels during decomposition.

In this paper, MVMD is employed to decompose the raw PV power series, aiming to mitigate the volatility of the generation data and thereby simplify the prediction task. The essential procedures of MVMD can be summarized as follows:

(1) For input data containing c channels of data, denoted as $x\left(t\right)=\left[x_{1}\left(t\right),x_{2}\left(t\right),\cdots ,x_{c}\left(t\right)\right]$. Suppose there are k multivariate modulation oscillations, such that:
  (1) $$x(t)=\sum_{k}u_{k}(t)$$
(2) The Hilbert-Huang transform is applied to each element of $u_{k}(t)$, denoted as $u_{+}^{k}(t)$, and then multi plied by the exponential term $e^{-j\omega_{k}t}$ to adjust it to the corresponding center frequency. The bandwidth of each mode $u_{k}(t)$ is estimated by using $\omega_{k}(t)$ as a harmonic mixer of $u_{+}^{k}(t)$, and then by the $L_{2}$ parameter of the gradient function of the harmonically transformed $u_{+}^{k}(t)$. MVMD constrains the decomposition such that the total bandwidth of the extracted modes is minimized, while ensuring that the resulting oscillatory components can faithfully reconstruct the original signal. Under this principle, the problem is formulated as a constrained variational optimization task, expressed as follows.
  (2) $$\underset{\left\{u_{k,c}\},\{\omega_{k}\right\}}{\min}\left\{\sum_{k}\sum_{c}{\left\|\partial_{t}[u_{k,c}^{+}(t)e^{-j\omega_{k}t}]\right\|}_{2}^{2}\right.$$
  (3) $$s.t.x_{c}(t)=\sum_{k}u_{k,c}(t)$$
(3) In solving multiple variational problems, the number of equations in the system of linear equations corresponds to the total number of channels, and accordingly, the augmented Lagrangian function is as follows.
  (4) $$L(\{u_{k,c}\},\{\omega_{k}\},\lambda_{c})=\alpha \sum_{k}\sum_{c}{\left\|\partial_{t}[u_{k,c}^{+}(t)e^{-j\omega_{k}t}]\right\|}_{2}^{2}+{\sum_{c}\left\|x_{c}\left(t\right)-\sum_{k}u_{k,c}\left(t\right)\right\|}_{2}^{2}+\sum_{c}\langle \lambda_{c}\left(t\right),x_{c}\left(t\right)-\sum_{k}u_{k,c}\left(t\right)\rangle$$
  where *λ* represents the Lagrangian multiplier introduced to enforce the constraint that the sum of the modes equals the original input signal.
(4) In order to solve this transformed unconstrained variational problem, alternate direction method of multipliers (alternate direction method of multipliers (ADMM)) is applied to realize the alternate updating, and then the decomposed signal components are obtained by taking the center frequency. The mode update is expressed as:
  (5) $${\hat{u}}_{k,c}^{l+1}(\omega )=\frac{{\hat{x}}_{c}(\omega )-\sum_{i\neq k}{\hat{u}}_{i,c}(\omega )+\frac{{\hat{\lambda }}_{c}(\omega )}{2}}{1+2\alpha {\left(\omega -\omega_{k}\right)}^{2}}$$
  The center frequency update obtained is expressed as:
  (6) $$\omega_{k}^{l+1}=\frac{\sum_{c}\int_{0}^{\infty }\omega {\left|{\hat{u}}_{k,c}(\omega )\right|}^{2}d\omega }{\sum_{c}\int_{0}^{\infty }{\left|{\hat{u}}_{k,c}(\omega )\right|}^{2}d\omega }$$

The signal’s frequency band is adaptively separated using the above update relation, resulting in k narrowband IMF components. Moreover, as MVMD can process multichannel data simultaneously, each channel yields the same number of IMFs, and the multivariate IMFs at the same level share identical frequency scales. This guarantees inter-channel frequency consistency and enhances the stability of signal analysis.

### 2.2. cINFO-GRU-ATT Model

#### 2.2.1. GRU Network

In PV power prediction, a substantial amount of data must be processed, and the gradient problem is susceptible to occurring during the training process, given the inherent volatility of photovoltaic data. GRU effectively addresses the gradient problem that arises in long sequences of traditional RNN (recurrent neural network) through its gating mechanism [37]. Furthermore, the GRU is capable of dynamically adjusting the amount of information that is retained and forgotten through the updating and resetting of the gate, which allows it to adapt more effectively to the temporal dynamics of the data.

**Figure 1:**
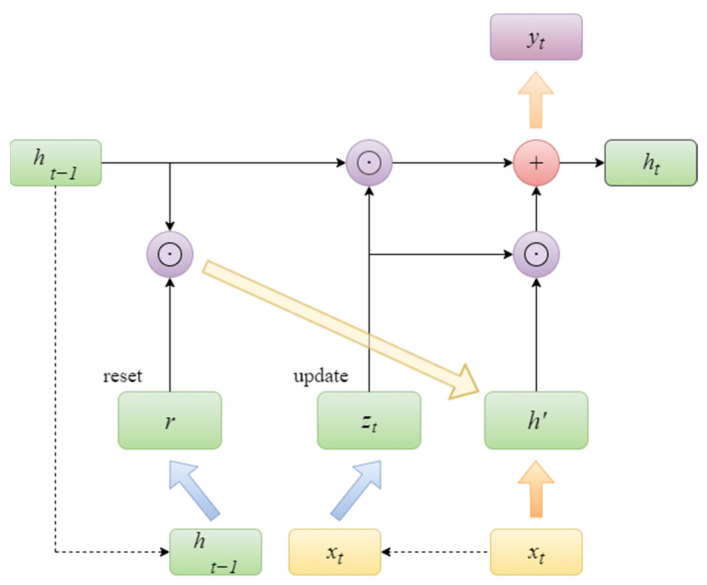
GRU unit structure.

Concurrently, GRU can markedly enhance the precision of prediction by efficiently discerning patterns and trends in historical data. In comparison to LSTM, GRU exhibits a more straightforward configuration and enhanced computational efficiency, which is a crucial attribute when processing extensive datasets. Accordingly, this paper employs GRU for the purpose of predicting PV power. The unit structure of GRU is illustrated in Figure 1.

The methodology employed for the prediction of PV power is as follows:

(1) Data Input: A set of input features, including historical power generation and meteorological data, should be utilized as input data.
(2) The initial state of the hidden layer is set.
(3) Time step cycling.

The following loop should be performed for each time step t (1 − T):

The gating states of the update and reset gates are initially determined by analyzing the last transmitted down state $h_{t-1}$ and the input $x_{t}$ of the current node.

(7) $$r_{t}=\sigma \left(W_{r}\cdot \left[h_{t-1},x_{t}\right]+b_{r}\right)$$

(8) $$z_{t}=\sigma \left(W_{z}\cdot \left[h_{t-1},x_{t}\right]+b_{z}\right)$$

The subsequent step is to utilize reset gating $r_{t}$ to obtain the data $h_{t-1}^{\prime }$ following the reset, and then splice it with $x_{t}$ to obtain $h_{t}^{\prime }$.

(9) $$h_{t-1}^{\prime }=r_{t}⊗h_{t-1}$$

(10) $$h_{t}^{\prime }=\tanh\left(W_{h}\cdot \left[h_{t-1}^{\prime },x_{t}\right]+b_{h}\right)$$

It is now necessary to update the memory using the technique of update gating $z_{t}$.

(11) $$h_{t}=z_{t}⊗h_{t-1}+\left(1-z_{t}\right)⊗h_{t}^{\prime }$$

In the case of update gating $z_{t}$ tending towards 1, the output is predominantly $h_{t-1}$ from the preceding moment. Conversely, when updating gating $z_{t}$ tending towards 0, the output is primarily $h_{t}^{\prime }$, that is to say, the new $h_{t}$.

#### 2.2.2. Attention Mechanisms

**Figure 2:**
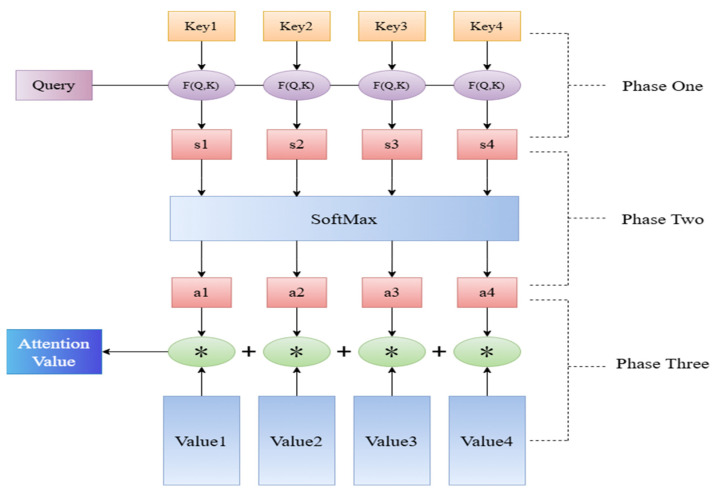
Structure of the Attention Mechanism.

In the context of recurrent neural networks, the passage of time inevitably results in the neuron states of earlier time information having smaller and smaller weights. This phenomenon, known as information forgetting, makes it increasingly challenging for the network to extract the features of the earlier time information, particularly when dealing with long time series data. The attention mechanism (AM) is designed to emulate the attention mechanism observed in the human visual and perceptual system. This mechanism enables the neural network to focus its attention on the most salient aspects of sequential data, thereby enhancing the model’s overall performance. By assigning different weights to input features, the attention mechanism helps the model focus on the most important factors, improving prediction accuracy. The structure of this mechanism is shown in Figure 2.

The process of attentional mechanisms can be described as follows:

(1) Calculate the degree of similarity (attention score) between the decoder state at the previous moment and the encoder output at each subsequent moment:
  (12) $$e_{t,i}=F\left(h_{i},s_{t-1}\right)$$
  where $h_{i}$ is the *i*-th output of the encoder; $s_{t-1}$ is the output state of the decoder at the moment t − 1; and F is the transformation function for computing the attention score.
(2) The attention scores calculated in the previous step are subjected to a softmax transformation, thereby obtaining their probability distribution:
  (13) $$\beta_{t}=soft\max\left(e_{t}\right)$$
  where $e_{t}$ is the number of attention components of the decoder to the encoder at moment t; and $\beta_{t}$ is the probability distribution of $e_{t}$.
(3) The attention vector $a_{t}$ is to be computed at moment t based on $\beta_{t}$ and the state of the full encoder:
  (14) $$a_{t}=\sum_{i=1}^{n}\beta_{t,i}h_{i}$$
(4) The attention vector should be combined with the input from the decoder to create a new input for decoding:
  (15) $$s_{t}=f\left(\left[c_{t},a_{t}\right],s_{t-1}\right)$$
  where $c_{t}$ is the input to the decoder at moment t; f is the transform function that computes the decoded input.

#### 2.2.3. Improved INFO Algorithm

The Vector weighted average algorithm is a population-based optimization algorithm that computes the weighted mean of a set of vectors in the search space. It does so by improving the weighted average method and updating the positions of the vectors, thereby forming a stronger, more robust structure. The three core processes of INFO [38] are rule updating, vector combination, and local search.

(I) Initialization stage

The INFO algorithm is constituted by the entirety of the $N_{p}$’s within the D-dimensional search region. In the initialization phase, two control parameters of INFO are introduced: the weighted average factor δ and the scale factor σ. These two factors are not subject to human adjustment and can be modified in real time according to the generation. A straightforward method employed by INFO to generate the initial vectors is known as random generation.

(II) Stage of updating the rules

The updating rules phase serves to enhance population diversity throughout the search process. This phase comprises two principal components. The initial stage of the mean-based approach commences with the generation of a random initial solution. Subsequently, a set of randomly selected vectors is updated with weighted average information, resulting in the generation of the subsequent solution. The second part incorporates a convergence acceleration technique to enhance the rate of convergence of the algorithm. The primary equation that defines the update rule phase is as follows:

The acceleration component (CA) enables the attainment of the optimal global position by guiding the current vector within the search space in accordance with the optimal vector.

(16) $$CA=randn\frac{\left(x_{bs}-x_{\alpha 1}\right)}{\left(f\left(x_{bs}\right)-f\left(x_{\alpha 1}\right)+\varepsilon \right)}$$

When rand < 0.5:

(17) $$z1_{l}^{g}=x_{l}^{g}+\sigma \times R+\frac{randn\left(x_{bs}-x_{\alpha 1}^{g}\right)}{f\left(x_{bs}\right)-f\left(x_{\alpha 1}^{g}\right)+1}$$

(18) $$z2_{l}^{g}=x_{bs}+\sigma \times R+\frac{randn\left(x_{\alpha 1}^{g}-x_{b}^{g}\right)}{f\left(x_{\alpha 1}^{g}\right)-f\left(x_{\alpha 2}^{g}\right)+1}$$

When rand ≥ 0.5:

(19) $$z1_{l}^{g}=x_{\alpha }^{g}+\sigma \times R+\frac{randn\left(x_{\alpha 2}^{g}-x_{\alpha 3}^{g}\right)}{f\left(x_{\alpha 2}^{g}\right)-f\left(x_{\alpha 3}^{g}\right)+1}$$

(20) $$z1_{l}^{g}=x_{bs}+\sigma \times R+\frac{randn\left(x_{\alpha 1}^{g}-x_{\alpha 2}^{g}\right)}{f\left(x_{\alpha 1}^{g}\right)-f\left(x_{\alpha 2}^{g}\right)+1}$$

In this context, the symbols $z1_{l}^{g}$ and $z2_{l}^{g}$ represent the new position vectors of the gth iteration, with $l=1,2,\cdots ,N_{p}$. The symbol σ denotes the scaling rate (scale factor) of the vector, while $f\left(x\right)$ signifies the fitness function of x. The vector $x_{bs}$, on the other hand, denotes the optimal solutions in the population of the gth generation. The symbol α represents a random distinct integer in the range [1, $N_{p}$], while randn denotes a random value of a standard normal distribution. Finally, the symbol α can be updated according to the exponential function.

(III) Vector combination stage

In the vector combination phase, INFO combines the two vectors $z1_{l}^{g}$ and $z2_{l}^{g}$, which were computed in the previous phase, with rand < 0.5 to generate a new vector $u_{l}^{g}$. This operator is utilized to enhance the local search of the vector, thereby facilitating the generation of a superior vector.

When rand1 < 0.5, rand2 < 0.5:

(21) $$u_{l}^{g}=z1_{l}^{g}+\mu \left|z1_{l}^{g}-z2_{l}^{g}\right|$$

When rand1 < 0.5, rand2 ≥ 0.5:

(22) $$u_{l}^{g}=z2_{l}^{g}+\mu \left|z1_{l}^{g}-z2_{l}^{g}\right|$$

When rand1 > 0.5:

(23) $$u_{l}^{g}=x_{l}^{g}$$

where $u_{l}^{g}$ is the new vector resulting from the combination of the *g*th generation vectors and μ=0.05×randn.

(IV) Local search stage

In vector-weighted optimization algorithms, an effective local search strategy is employed to prevent the algorithm from falling into a local optimum. This approach facilitates the convergence of the operator to a globally optimal solution. When rand1 < 0.5 and rand is a random value of [0, 1], a new vector is generated.

When rand1 < 0.5, rand2 < 0.5:

(24) $$u_{l}^{g}=x_{bs}+randn\left(R+randn\left(x_{bs}^{g}-x_{\alpha 1}^{g}\right)\right)$$

When rand1 < 0.5, rand2 ≥ 0.5:

(25) $$u_{l}^{g}=x_{rand}+randn\{R+randn\left(v1\times x_{bs}-v2\times x_{rand}\right)\}$$

(26) $$x_{rand}=\phi \times x_{avg}+\left(1-\phi \right)\times \left(\phi \times x_{bt}+\left(1-\phi \right)x_{bs}\right)$$

where ϕ is random value of [0, 1]; $x_{rand}$ is the combination of $x_{avg}$, $x_{bt}$, and $x_{bs}$ into a new solution.

To improve the performance of the INFO optimization algorithm, this study proposes an enhanced version.

In this study, the INFO optimization algorithm is combined with the CPO algorithm. The INFO algorithm excels at global exploration and local exploitation within the search space, using weight factors to guide solution updates and balance global and local search. However, it may exhibit slow convergence in certain scenarios. In contrast, the CPO algorithm emphasizes maintaining solution diversity, employing various “defense mechanisms” to escape local optima and dynamically adjusting the search range based on population size, thereby improving convergence speed in later stages. The two optimization algorithms complement each other at the technical level and can improve the performance of the algorithm in general.

In the initialization phase, this paper chooses to replace the original random stochastic initialization population with a Latin hypercubic initialization population. In Latin hypercube sampling, each dimension is divided into equal-width intervals, and a sample point is randomly selected in each interval. The selected points are then mapped to the actual search space. The precise expression is as follows:

(27) $$x_{i,j}=\frac{P_{i,j}+U_{i,j}}{n}$$

(28) $$X=lb+X_{std}\times \left(ub-lb\right)$$

where $P_{i,j}$ is the interval number taking the value of (0, n − 1); $U_{i,j}$ is a random number uniformly distributed in [0, 1); $X_{std}$ is the standardized sample matrix generated by the LHS; and lb and ub are the lower and upper bound vectors, respectively.

**Figure 3:**
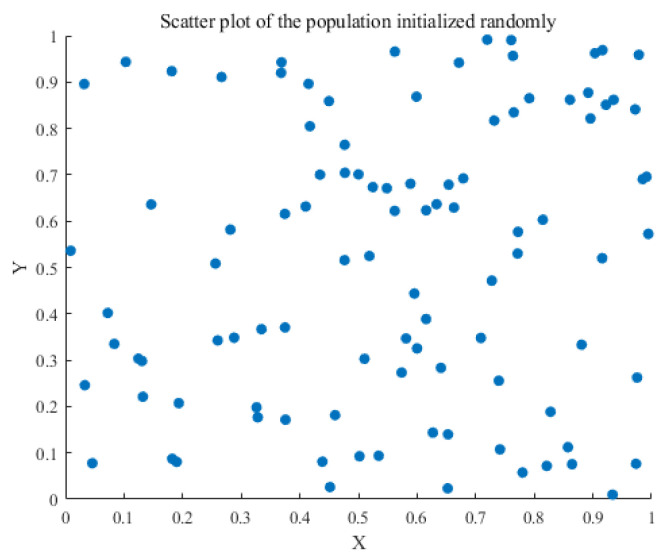
Random initialization.

**Figure 4:**
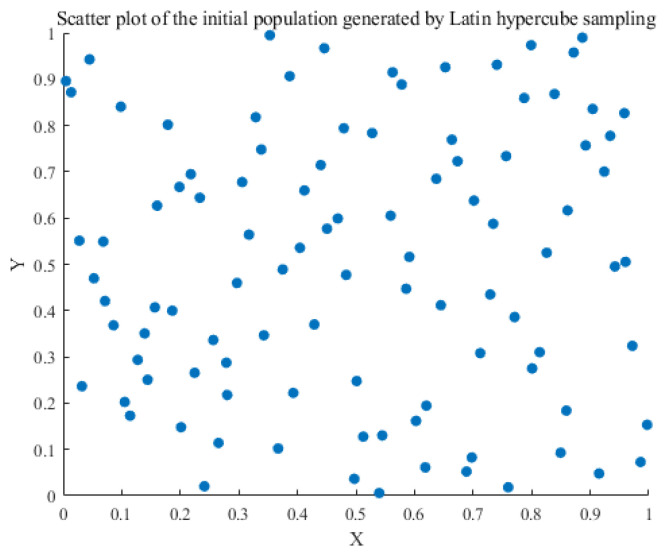
Latin hypercube sampling initialization.

The LHS guarantees that the sample points are distributed uniformly across all dimensions, thereby circumventing the issue of sample point concentration in specific regions and sparsity in others. This even distribution contributes to an increase in population diversity while providing more useful information in the initial stages of optimization, thus enhancing the efficiency and convergence of the global search. Figure 3 and Figure 4 illustrate the scatter plots of the initialized population, generated through random initialization and Latin hypercube initialization.

The update rule of INFO is used in the update phase of the solution, along with the defense mechanism of CPO for local search.

(1) Randomly select three solutions in the population: A(a), A(b), A(c).
(2) Calculation of the fitness difference:

Fitness difference:

(29) $$MM=\left[M\left(a\right)-M\left(b\right),M\left(a\right)-M\left(c\right),M\left(b\right)-M\left(c\right)\right]$$

Weighting:

(30) $$W\left(j\right)=\cos\left(MM\left(j\right)+\pi \right)\cdot \exp\left(-\frac{MM\left(j\right)}{omg}\right)$$

(3) Solution Updates:

Synthesize the amount of updates to the current solution:

(31) $$WM1=\frac{del\cdot \sum_{j-1}^{3}W\left(j\right)\cdot \left(X\left(a,:\right)-X\left(b,:\right)\right)}{Wt+1}$$

(32) $$WM2=\frac{del\cdot \sum_{j-1}^{3}W\left(j\right)\cdot \left(Best\_X-Worst\_X\right)}{Wt+1}$$

Combine the two updating methods by means of a random number *r*:

(33) $$MeanRule=r\cdot WM1+\left(1-r\right)\cdot WM2$$

(4) Combining CPO defense mechanisms enhances exploration through perturbation:

(34) $$z1=X\left(i,:\right)+\sigma \cdot \left(rand\cdot MeanRule\right)+\frac{randn\cdot \left(Best\_X-X\left(a,:\right)\right)}{M_{Best}-M\left(a\right)+1}$$

(35) $$z2=Best\_X+\sigma \cdot \left(rand\cdot MeanRule\right)+\frac{randn\cdot \left(X\left(a,:\right)-X\left(b,:\right)\right)}{M\left(a\right)-M\left(b\right)+1}$$

(5) Introducing a localized search mechanism:

(36) $$X_{avg}=\frac{X\left(a,:\right)+X\left(b,:\right)+X\left(c,:\right)}{3}$$

(37) $$X_{rnd}=\phi \cdot X_{avg}+\left(1-\phi \right)\cdot \left(\phi \cdot Better\_X+\left(1-\phi \right)\cdot Best\_X\right)$$

Dynamic weight calculation, perturbation mechanism and local search are introduced in order to enhance the global and local exploration capability of the algorithm.

## 3. Research Framework

**Figure 5:**
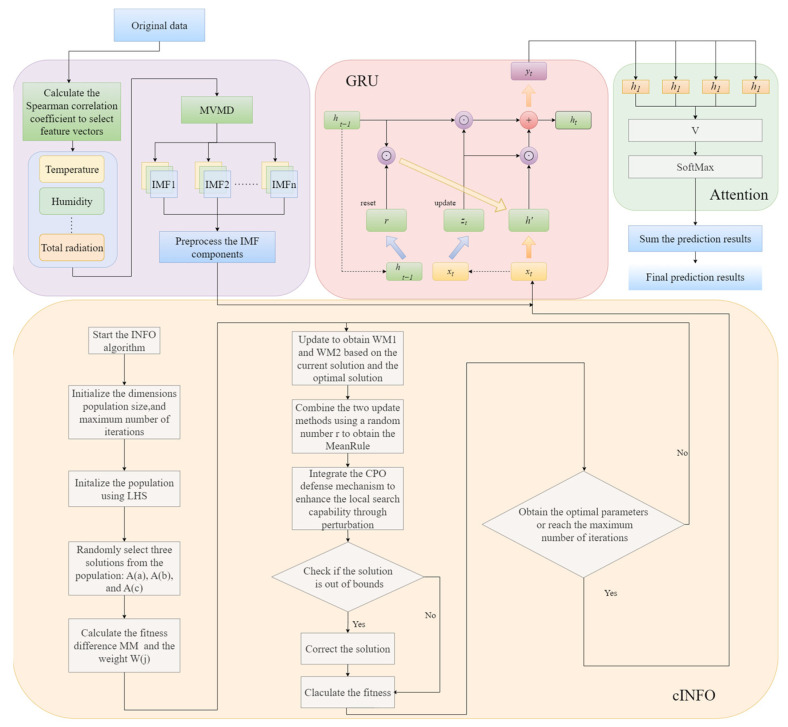
Schematic Diagram of an Ensemble Prediction Model.

The fundamental tenets of the MVMD-cINFO-GRU-ATT PV power prediction model, as elucidated in this paper, are illustrated in Figure 5.

1. A corpus of historical datasets pertaining to PV power plants has been assembled, comprising both PV data and meteorological data. The ratio of the training set to the test set of the data utilized in the model presented in this paper is 8:2.
2. The Spearman correlation coefficients of all meteorological factors were calculated, and the input meteorological factors were selected based on the correlation coefficients. The selected factors were temperature, humidity, total radiation, direct radiation, and diffuse radiation.
3. The historical PV power generation data were decomposed using MVMD to extract the corresponding modal components.
4. The GRU-ATT parameters are optimized using cINFO, thereby enhancing the model’s performance. The MVMD-decomposed PV data and correlation-screened meteorological data are employed as inputs, and the trained cINFO-GRU-ATT is utilized as a prediction model to forecast the PV power in the subsequent time period.
5. The output prediction components are then superimposed to obtain the final prediction results. The results of the prediction are then summarized and analyzed in order to verify the feasibility and superiority of the model proposed in this paper.
6. In this study, data from three weather conditions (sunny, cloudy, and rainy) were selected and predicted to assess the generalizability of the model.

## 4. Experiment and Discussion

### 4.1. Data Introduction

This study utilized an experimental PV dataset from the Desert Knowledge Australia Solar Centre (DKASC) collected in 2016, recording power generation at site three (4.95 kW) in Alice Springs [39]. The PV power output and corresponding meteorological data (e.g., solar irradiance, temperature) are sampled at a 5 min resolution. The installation parameters of the PV system are listed in Table 2. Historical meteorological data were derived from short-term forecasts based on records from the Alice Springs weather station.

### 4.2. Dataset Processing

#### 4.2.1. Abnormal Data Processing and Data Normalization

Handling outliers and missing values in the dataset can mitigate their adverse effects on the model’s training process. In this study, the K-approximation method (KNN) was employed to replace and fill in outliers and missing data in the experimental dataset.

Data normalization, on the other hand, is to turn the data into a consistent range and ensure the correlation between the data. In this paper, minimum-maximum normalization is used for normalization, and all feature data are normalized and then input into the model.

#### 4.2.2. Dataset Partitioning and Feature Vector Filtering

The dataset includes nine meteorological factors relevant to PV power generation: wind speed, temperature (°C), relative humidity, horizontal irradiance, diffuse horizontal irradiance, tilted irradiance, diffuse tilted irradiance, wind direction, and daily rainfall. The selection of input meteorological features can significantly influence the model’s predictive performance. In this study, a Gaussian mixture model was first applied to classify the dataset into three weather types: sunny, cloudy, and rainy. Horizontal irradiance, diffuse horizontal irradiance, temperature, daily rainfall, and relative humidity were chosen as features for clustering, as horizontal irradiance, diffuse irradiance, and temperature exhibit greater variability on cloudy and rainy days compared to sunny conditions. Also, daily rainfall and relative humidity can be well differentiated between rainy days and other weather types. Parameters (mean, variance and mixing coefficients) of each Gaussian distribution were estimated using a Gaussian mixture model through the expectation maximization (EM) algorithm. The weather type was determined by using these parameters to classify the data points into the most likely Gaussian distribution.

**Figure 6:**
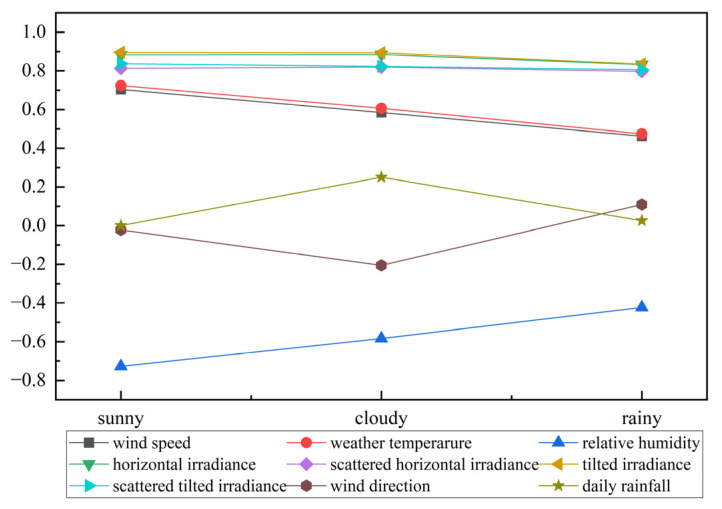
Figure of Spearman correlation coefficients.

Then, the Spearman correlation coefficients between the meteorological factors and the PV power were calculated under three different weather types, and the correlation coefficients under the three weather types are tabulated in Table 3 and Figure 6. The Spearman correlation coefficients were not calculated for sunny days because the daily rainfall was zero. Finally, horizontal irradiance, scattered horizontal irradiance, tilted irradiance, tilted horizontal irradiance, weather temperature and relative humidity were selected as input eigenvectors based on the correlation during sunny days. Horizontal irradiance, scattered horizontal irradiance, tilted irradiance, tilted horizontal irradiance, weather temperature and wind custom were selected as input eigenvectors for both cloudy and rainy days.

### 4.3. Prediction Accuracy Evaluation Index

To assess the predictive accuracy of PV power, this study uses mean absolute error (MAE), and root mean square error (RMSE) as evaluation metrics. The formulas for these metrics are as follows:

(38) $$MAE=\frac{1}{n}\sum_{i=1}^{n}\left|{\hat{y}}_{i}-y_{i}\right|$$

(39) $$RMSE=\sqrt{\frac{1}{n}\sum_{i=1}^{n}{\left(y_{i}-{\hat{y}}_{i}\right)}^{2}}$$

where $y_{i}$ is the *i*th true value; ${\hat{y}}_{i}$ is the *i*th predicted value.

### 4.4. Comparative Models

To verify the effectiveness of the proposed MVMD-cINFO-GRU-ATT model, six comparison models were established: LSTM, GRU, GRU-ATT, INFO-GRU-ATT, cINFO-GRU-ATT, and MVMD-INFO-GRU-ATT. First, LSTM and GRU were compared, showing that GRU outperforms LSTM under complex weather conditions, thus serving as the base model for the proposed combined prediction framework. Next, GRU-ATT was compared with GRU to demonstrate how the attention mechanism enables the model to focus on key data patterns, improving its ability to capture data variations. INFO-GRU-ATT was then compared with GRU-ATT, highlighting that hyperparameter optimization enhances prediction accuracy. The cINFO-GRU-ATT model was compared with INFO-GRU-ATT to illustrate that the cINFO optimization algorithm combined with CPO further improves GRU performance. Subsequently, MVMD-INFO-GRU-ATT was compared with INFO-GRU-ATT, showing that multivariate variational mode decomposition reduces historical data volatility, allowing the model to better capture fluctuations and achieve higher prediction accuracy. Finally, MVMD-cINFO-GRU-ATT was compared with MVMD-INFO-GRU-ATT, confirming that hyperparameter optimization via the proposed cINFO algorithm further enhances prediction accuracy even when using decomposed input components.

### 4.5. Parameter Setting

To evaluate the performance of the proposed MVMD-cINFO-GRU-ATT model, comparative experiments were conducted using the following models: LSTM, GRU, GRU-ATT, INFO-GRU-ATT, cINFO-GRU-ATT, MVMD-INFO-GRU-ATT, and MVMD-cINFO-GRU-ATT. All models were used to predict PV power under three different weather conditions. To ensure a fair comparison, model parameters were kept consistent across experiments. This experiment is based on MATLAB R2022b platform, Intel Core i7-12700H processor, 32 GB RAM, and an NVIDIA RTX 4060 GPU.

As shown in Table 4, max_iter is the maximum number of iterations, K is the number of modal decompositions, L is the L2 regularization coefficient, R is the optimal learning rate, Hidden_nodes is the optimal number of nodes in the hidden layer, and N is the number of neural network layers. Due to different sun levels, the parameter settings are different in different weather conditions. The proposed model uses a maximum of 300 iterations, with a modal decomposition number of 6 and L2 regularization coefficients of 1 × 10^−6^, 1 × 10^−6^, and 1 × 10^−6^, respectively. The optimal learning rates were 0.0093, 0.0078, and 0.007, respectively. The optimal number of hidden tier nodes is 93, 67, and 100, respectively. The number of neural network layers is 7, including: input layer, GRU layer, dropout layer, selfAttentionLayer, ReLU activation layer, fullyConnectedLayer, and regressionLayer.

### 4.6. Results Analysis

**Figure 7:**
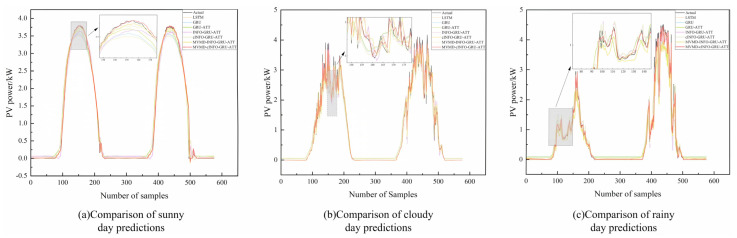
Comparison of prediction results.

**Figure 8:**
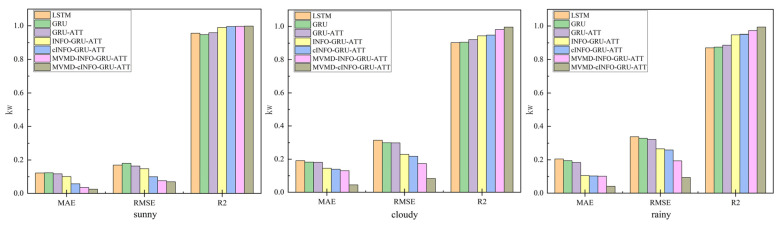
Error plots.

In this study, three different weather types were selected for prediction and two days for each weather type were selected for visualization and analysis. The graphs of the prediction results for the three different weather types are shown in Figure 7. The error plot and error table are shown in Figure 8 and Table 5. From the prediction comparison graphs and errors, it can be seen that the combined prediction model proposed in this paper has the most accurate results.

Predictions from all models are applicable across the three weather types, yet notable differences in accuracy exist. For each model, prediction performance under rainy conditions is worse than under sunny or cloudy conditions, as rainfall and low irradiance during rainy days significantly affect PV power generation and introduce greater volatility in historical data.

Single models such as LSTM and GRU perform better under sunny conditions due to stronger irradiance and lower variability in historical data. Introducing the attention mechanism in GRU-ATT reduces MAE, and RMSE by 4.18%, 3.25% and 5.11%, 9.00%, respectively, and improves R^2^ by 0.3% and 1.12% compared to LSTM and GRU. Applying the INFO optimization algorithm for GRU hyperparameters further reduces MAE, and RMSE of INFO-GRU-ATT by 13.8%, 18.3%, and 9.65% relative to GRU-ATT, with a 3.36% increase in R^2^. In this study, the INFO algorithm was enhanced and combined with the CPO algorithm to form cINFO. Using cINFO for GRU hyperparameter optimization, the MAE, and RMSE of cINFO-GRU-ATT decreased by 43.21%, 54.79%, and 32.72%, respectively, compared to INFO-GRU-ATT, while R^2^ increased by 0.53%. Decomposition of historical data using MVMD under sunny day type and prediction of components before superposition yielded the final prediction, and the MAE and RMSE of the MVMD-INFO-GRU-ATT model were reduced by 64.91%, 73.97%, and 48.75%, respectively, as compared to those of the INFO-GRU-ATT model; and the R2 was improved by 0.62%. In the case of using MVMD decomposition, using cINFO for hyperparameter optimization of GRU before prediction, the model MVMD-cINFO-GRU-ATT model reduces the MAE and RMSE by 29.67%, 15.79% and 8.58%, respectively, as compared to the MVMD-INFO-GRU-ATT model; the R2 improves 0.14%.

Under cloudy weather types, the prediction effect of LSTM is reduced relatively substantially compared to that in sunny weather. This is because the irradiance decreases and the temperature decreases in cloudy weather, and the volatility of the PV data increases. The GRU model has a relatively simple structure, is easier to train with fewer parameters in the complex mode, and is able to quickly adapt to the different weather changes, and can better capture these short-term characteristics of the meteorological factors. Compared with the LSTM model, the GRU model achieves reductions in MAE, and RMSE of 4.75%, 8.65%, and 4.43%, respectively, with an R^2^ increase of 0.23%. Incorporating the attention mechanism further decreases the MAE, and RMSE of GRU-ATT by 0.49%, 0.78%, and 0.37%, respectively, relative to GRU, while R^2^ improves by 1.59%. Following hyperparameter optimization using the INFO algorithm, INFO-GRU-ATT reduces MAE, and RMSE by 19.90%, 41.08%, and 23.24%, respectively, compared to GRU-ATT, with R^2^ rising by 2.32%. Further optimization with the cINFO algorithm lowers the MAE, and RMSE of cINFO-GRU-ATT by 4.41%, 9.90%, and 5.02%, respectively, over INFO-GRU-ATT, and improves R^2^ by 0.41%. Through decomposition of historical data using MVMD under multi-cloud type and prediction of components before superposition to get the final prediction results, the MAE and RMSE of the MVMD-INFO-GRU-ATT model compared to the INFO-GRU-ATT model were reduced by 10.32%, 43.05%, and 24.56%, respectively; and the R2 was improved by 3.69%. In the case of using MVMD decomposition, using cINFO for hyper-parameter optimization of GRU before prediction, the model MVMD-cINFO-GRU-ATT model reduces the MAE and RMSE by 65.92%, 76.92% and 51.94%, respectively, compared to the MVMD-INFO-GRU-ATT model; and the R2 improves by 1.42%.

Under rainy conditions, both LSTM and GRU show greater performance differences than under cloudy weather, as rainy days feature the lowest irradiance and temperature, along with higher humidity, making PV data highly volatile. Nevertheless, the GRU model reduces MAE and RMSE by 4.83% and 2.73%, respectively, compared to LSTM, with an R^2^ improvement of 0.44%. It indicates that GRU has better ability to capture features under complex weather types and can better adapt to weather changes. With the introduction of the attention mechanism, the GRU-ATT model reduces MAE and RMSE by 5.69% and 2.22%, respectively, compared to GRU, with an R^2^ improvement of 1.16%. Following hyperparameter optimization using the INFO algorithm, INFO-GRU-ATT achieves reductions of 42.79% and 17.08% in MAE and RMSE relative to GRU-ATT, along with a 6.19% increase in R^2^. Further optimization using the cINFO algorithm decreases MAE and RMSE of cINFO-GRU-ATT by 2.00% and 2.63%, respectively, compared to INFO-GRU-ATT, with an R^2^ gain of 0.27%. Through decomposition of the historical data using MVMD under multi-cloud type and prediction of components before superposition to achieve the final prediction results, the MAE and RMSE of the MVMD-INFO-GRU-ATT model compared to the INFO-GRU-ATT model were reduced by 3.42% and 27.13%, respectively; and the R2 was improved by 2.44%. In the case of using MVMD decomposition, using cINFO for hyperparameter optimization of GRU before prediction, the model MVMD-cINFO-GRU-ATT model reduces the MAE and RMSE by 59.45% and 51.62%, respectively, compared to the MVMD-INFO-GRU-ATT model; and the R2 improves by 2.11%.

To further assess the performance of the cINFO optimization algorithm proposed in this paper, its performance is evaluated using test functions and compared with other optimization algorithms, and the comparison results are shown in.

**Figure 9:**
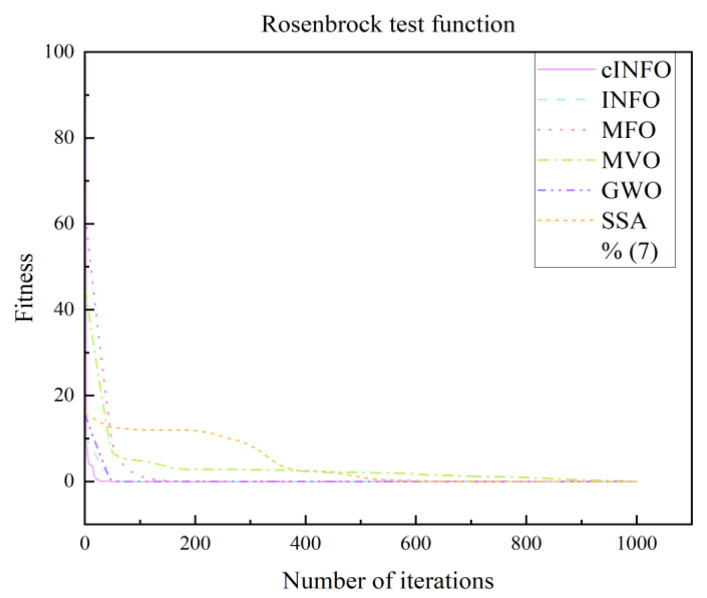
Rosenbrock test function.

**Figure 10:**
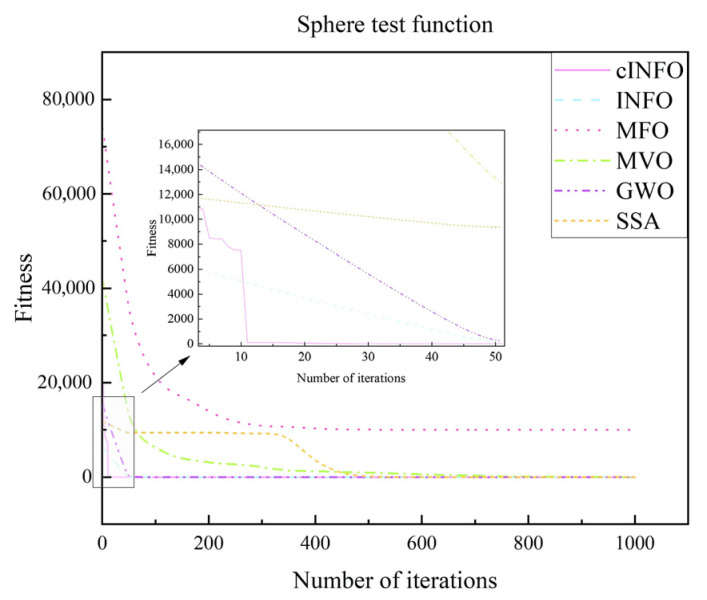
Sphere test function.

Figure 9 presents the performance evaluation of various optimization algorithms using the Rosenbrock test function, while Figure 10 shows the results using the Sphere test function. As illustrated in both figures, the proposed cINFO algorithm exhibits faster convergence and a stronger capability to locate the global optimum compared with other optimization algorithms, including the original INFO algorithm.

## 5. Conclusions

With the growing concerns over energy scarcity and global warming, PV power generation is increasingly being adopted worldwide. Accurate PV power forecasting supports more efficient power scheduling and improves both the economic performance and stability of the power grid. To enhance prediction accuracy, this study proposes a hybrid GRU-ATT model integrating improved MVMD and cINFO, which has shown high predictive performance in experimental evaluations. The main research contributions are summarized as follows:

(1) Utilize the decomposition algorithm to decompose the historical data, obtain the sub-sequence, independently predict the sub-sequence, and superimpose the prediction results to derive the final prediction results. This process serves to reduce the volatility of historical data while simultaneously enhancing the accuracy of the resulting prediction.
(2) The INFO algorithm has been enhanced by the introduction of several new technologies. These include the use of Latin super cubic sampling to initialize the population, combined with the defense mechanism of the CPO optimization algorithm to enhance the exploration ability. This approach not only increases overall diversity but also strengthens both the global and local search abilities of the algorithm, helping to avoid local optima. Furthermore, the improved cINFO algorithm is integrated with GRU to optimize its parameters, enhancing overall model performance. An attention mechanism is also incorporated, allowing the model to more effectively capture salient sequence information and thereby improve the prediction accuracy of the combined model.
(3) The review demonstrates that the proposed combined prediction model achieves both high accuracy and strong generalizability, providing improved support for the scheduling and decision-making of DPV power generation systems.

While the model presented in this paper demonstrably enhances the precision of PV power forecasts, its applicability is constrained by two key limitations: the exclusive reliance on the single-site dataset and simplified meteorological processing that incorporates only five basic meteorological variables. This narrow scope omits critical factors such as cloud dynamics, atmospheric conditions, and non-meteorological influences, thereby restricting the model’s generalizability across diverse climatic regions and complex real-world scenarios where these variables significantly impact generation volatility. Future work will implement multi-site validation and integrate advanced meteorological processing to enhance cross-regional robustness.

## Figures and Tables

**Table 1:** Comparative Analysis of Representative Limitations in PV Forecasting Models.

Model Category	Specific Methods	Limitations
Signal Decomposition	VMD	Parameter sensitivity (mode number K/penalty factor α requires extensive tuning); multi-stage decomposition generates excessive subsequences, reducing efficiency.
Deep Learning Models	LSTM	Prone to overfitting during training; high parameter count increases computational cost.
Hybrid Model	Multi-decomposition (e.g., VMD-CEEMD-SSA)	Exponential increase in subsequences from multi-stage decomposition severely impacts computational efficiency and risks reconstruction errors.
Hybrid Model	Multi-model integration	Complex hyperparameter optimization; low synergy efficiency between modules.

**Table 2:** PV plants parameters.

Parameter	Value
Array Rating/kW	4.95
Panel Rating/W	165
Number of Panels	30
Panel Type	BP 3165
Array Area/m^2^	37.75
Inverter Size/Type	6 kW, SMA SMC 6000 A
Array Tilt/Azimuth	Tilt = 20, Azimuth = 0 (Solar North)

**Table 3:** Table of Spearman correlation coefficients.

Weather	Meteorological Factors	Correlation Coefficient
Sunny	wind speed	0.7042
	weather temperature (degrees Celsius)	0.7245
	relative humidity	−0.7276
	horizontal irradiance	0.8827
	scattered horizontal irradiance	0.8128
	tilted irradiance	0.8961
	scattered tilted irradiance	0.8371
	wind direction	−0.0221
	daily rainfall	NaN
Cloudy	wind speed	0.5841
	weather temperature (degrees Celsius)	0.6068
	relative humidity	−0.58301
	horizontal irradiance	0.8843
	scattered horizontal irradiance	0.8197
	tilted irradiance	0.8938
	scattered tilted irradiance	0.8228
	wind direction	−0.2057
	daily rainfall	0.2500
Rainy	wind speed	0.4623
	weather temperature (degrees Celsius)	0.4749
	relative humidity	−0.4234
	horizontal irradiance	0.8323
	scattered horizontal irradiance	0.7965
	tilted irradiance	0.8350
	scattered tilted irradiance	0.8056
	wind direction	0.1089
	daily rainfall	0.0253

**Table 4:** Model parameter table.

Weather	Model	Parameter Setting
Sunny	LSTM	max_iter = 300, learning rate = 0.01, Hidden_nodes = 10, N = 5
	GRU	max_iter = 300, learning rate = 0.01, Hidden_nodes = 10, N = 5
	GRU-ATT	max_iter = 300, learning rate = 0.01, Hidden_nodes = 10, N = 7
	INFO-GRU-ATT	max_iter = 300, L = 1 × 10^−6^, R = 0.0078, Hidden_nodes = 47, N = 7
	cINFO-GRU-ATT	max_iter = 300, L = 8.791 × 10^−4^, R = 5.189 × 10^−4^, Hidden_nodes = 100, N = 7
	MVMD-INFO-GRU-ATT	max_iter = 300, L = 2.078 × 10^−5^, R = 0.0035, Hidden_nodes = 91, N = 7, K = 6
	MVMD-cINFO-GRU-ATT	max_iter = 300, L = 1 × 10^−6^, R = 0.0093, Hidden_nodes = 93, N = 7, K = 6
Cloudy	LSTM	max_iter = 300, learning rate = 0.01, Hidden_nodes = 10, N = 5
	GRU	max_iter = 300, learning rate = 0.01, Hidden_nodes = 10, N = 5
	GRU-ATT	max_iter = 300, learning rate = 0.01, Hidden_nodes = 10, N = 7
	INFO-GRU-ATT	max_iter = 300, L = 1 × 10^−6^, R = 9.07 × 10^−4^, Hidden_nodes = 97, N = 7
	cINFO-GRU-ATT	max_iter = 300, L = 1 × 10^−6^, R = 0.0081, Hidden_nodes = 98, N = 7
	MVMD-INFO-GRU-ATT	max_iter = 300, L = 1 × 10^−6^, R = 0.01, Hidden_nodes = 29, N = 7, K = 6
	MVMD-cINFO-GRU-ATT	max_iter = 300, L = 1 × 10^−6^, R = 0.0078, Hidden_nodes = 67, N = 7, K = 6
Rainy	LSTM	max_iter = 300, learning rate = 0.01, Hidden_nodes = 10, N = 5
	GRU	max_iter = 300, learning rate = 0.01, Hidden_nodes = 10, N = 5
	GRU-ATT	max_iter = 300, learning rate = 0.01, Hidden_nodes = 10, N = 7
	INFO-GRU-ATT	max_iter = 300, L = 1.027 × 10^−6^, R = 0.0029, Hidden_nodes = 10, N = 7
	cINFO-GRU-ATT	max_iter = 300, L = 1 × 10^−6^, R = 0.0093, Hidden_nodes = 30, N = 7
	MVMD-INFO-GRU-ATT	max_iter = 300, L = 1.489 × 10^−6^, R = 0.0093, Hidden_nodes = 100, N = 7, K = 6
	MVMD-cINFO-GRU-ATT	max_iter = 300, L = 1 × 10^−6^, R = 0.007, Hidden_nodes = 100, N = 7, K = 6

**Table 5:** Error Comparison.

Weather	Model	MAE	RMSE	R^2^	Computational Time (s)
Sunny	LSTM	0.1221	0.1692	95.52%	2232.56
	GRU	0.1233	0.1799	94.75%	2156.32
	GRU-ATT	0.1170	0.1637	95.81%	2354.55
	INFO-GRU-ATT	0.1009	0.1479	99.03%	2659.03
	cINFO-GRU-ATT	0.0573	0.0995	99.56%	2703.25
	MVMD-INFO-GRU-ATT	0.0354	0.0758	99.65%	2756.23
	MVMD-cINFO-GRU-ATT	0.0249	0.0693	99.79%	2658.42
Cloudy	LSTM	0.1914	0.3136	90.32%	1604.65
	GRU	0.1823	0.2997	90.55%	1535.25
	GRU-ATT	0.1814	0.2986	92.14%	1603.76
	INFO-GRU-ATT	0.1453	0.2292	94.46%	1985.25
	cINFO-GRU-ATT	0.1389	0.2177	94.87%	1925.36
	MVMD-INFO-GRU-ATT	0.1303	0.1729	98.15%	2204.53
	MVMD-cINFO-GRU-ATT	0.0444	0.0831	99.57%	2145.33
Rainy	LSTM	0.2049	0.3374	87.01%	1325.33
	GRU	0.1950	0.3282	87.45%	1278.79
	GRU-ATT	0.1839	0.3209	88.61%	1326.88
	INFO-GRU-ATT	0.1052	0.2661	94.80%	1575.26
	cINFO-GRU-ATT	0.1031	0.2591	95.07%	1523.23
	MVMD-INFO-GRU-ATT	0.1016	0.1939	97.24%	1835.26
	MVMD-cINFO-GRU-ATT	0.0412	0.0938	99.35%	1756.25

## Data Availability

The original contributions presented in this study are included in the article. Further inquiries can be directed to the corresponding author.
